# Case of Abscess of the Liver, Consequent to Injury of the Head

**Published:** 1814-08

**Authors:** M. Reynaud


*For the Medical and Physical Journal.*

### *Case of Abscess of the Liver, consequent to Injury of the Head; by* M. Reynaud.

ON the 15th of March last, Louis Billard was at work in the hold of the ship Hannibal, and, whilst in a sitting posture, a wedge of wood three pounds in weight fell from the height of fifteen feet on his head, which produced a contused wound, about an inch and half long, across the posterior and superior right parietal bone, penetrating no deeper than the hairy scalp, and from which neither vertigo nor insensibility followed. He was, however, taken to the hospital, bled, and put on low diet. On the 25th the wound healed; and on the 27th he was to have been discharged, but when M. Reynaud visited him on that morning, he had considerable fever, thirst, furred and clammy tongue, bitter taste in the mouth, nausea, disposition to vomit, dry hot skin, pain across the forehead. M. R. conceiving it to be a bilious attack, gave him *barley water with a grain of emetic tartar*, which produced vomiting, but did not relieve the nausea.

28th.—Symptoms were the same. Medicine continued.

29th.—Tongue dry; great thirst; bowels constipated; frequent pulse; *pain in right hypochondrium, extending to the right shoulder*; eyes of a yellow tinge, as was also the whole surface of the body; stomach rejected every thing, and the man frequently produced vomiting by introducing his fingers into his throat. Lemonade and whey were prescribed; two emollient enemas and a large poultice to the seat of pain.

30th.—Symptoms the same: *great pain experienced in breathing*; pulse small. *Four leeches* were applied to the right right hypochondrium, and the bleeding encouraged for an hour, after emollient drinks and enemas were given. In the evening he was a little better.

31st.—Great oppression; sense of suffocation; could only rest in an upright position; small weak pulse; a sensible elevation at the right hypochondrium; emollients continued; tumour poulticed; he vomited every thing.

April 1st.—Tongue dry, yellow; great pain on the right side; great difficulty in breathing. Slight relief was experienced from the warm bath; prescriptions continued.

The patient's sufferings rapidly increased till the 3d, when the disease assumed a most afflicting appearance: he was in inexpressible agitation, breathed with the greatest difficulty, and the pulse was scarcely to be felt. In the evening he sunk (under this murderous treatment!—*Translator*).

On examination after death, nothing preternatural was discovered in the head. The liver was of an enormous size, turgid; and the gall-bladder filled with bile. The large lobe adhered in many places to the diaphragm, and three abscesses were found in it, the largest of which was close to the ligamentum coronarium. There was no communication from the liver to the thorax. The stomach was inflamed, and contained a little grumous blood. The other abdominal viscera were in their natural state.

The right cavity of the thorax contained about a pint and a half of a fluid similar in kind to the matter of the abscesses of the liver. The right lung had numerous adhesions to the diaphragm; the left lung retained its natural colour, and the adhesions of former inflammations. There was no disease about the heart.—*Journale Generale de Med.*

